# Computer Vision Technology for Monitoring of Indoor and Outdoor Environments and HVAC Equipment: A Review

**DOI:** 10.3390/s23136186

**Published:** 2023-07-06

**Authors:** Bin Yang, Shuang Yang, Xin Zhu, Min Qi, He Li, Zhihan Lv, Xiaogang Cheng, Faming Wang

**Affiliations:** 1 School of Energy and Safety Engineering, Tianjin Chengjian University, Tianjin 300384, China; binyang@tcu.edu.cn (B.Y.); 18202507201@163.com (S.Y.); xinzhu196@163.com (X.Z.); qimin813@163.com (M.Q.); lihe19981998@163.com (H.L.); 2 Department of Game Design, Faculty of Arts, Uppsala University, SE-62167 Uppsala, Sweden; 3 College of Telecommunications and Information Engineering, Nanjing University of Posts and Telecommunications, Nanjing 210042, China; chengxg@njupt.edu.cn; 4 Department of Biosystems, KU Leuven, 3001 Leuven, Belgium; faming.wang@kuleuven.be

**Keywords:** computer vision, behavior patterns, remote sensing, equipment health monitoring, fault diagnosis and detection, non-contact measurement

## Abstract

Artificial intelligence technologies such as computer vision (CV), machine learning, Internet of Things (IoT), and robotics have advanced rapidly in recent years. The new technologies provide non-contact measurements in three areas: indoor environmental monitoring, outdoor environ-mental monitoring, and equipment monitoring. This paper summarizes the specific applications of non-contact measurement based on infrared images and visible images in the areas of personnel skin temperature, position posture, the urban physical environment, building construction safety, and equipment operation status. At the same time, the challenges and opportunities associated with the application of CV technology are anticipated.

## 1. Introduction

### 1.1. Research Background

A comfortable indoor and outdoor environment and efficiently operating HVAC equipment are essential for human health, productivity growth, and energy savings. Traditional indoor and outdoor environment monitoring, as well as HVAC equipment condition monitoring, are limited in some way by the technical constraints of the time and space. With the technological advancements in artificial intelligence, these three fields are bound to break free from their original constraints and open up new avenues for development [1]. The research background of the aforementioned three fields is described below.

#### 1.1.1. Indoor Environment Monitoring

A comfortable and healthy indoor environment is critical for occupant health and productivity. With the rise of the concept of “human-centered” buildings in recent years, indoor environmental control proposes starting from people’s actual needs, including the real-time measurement of people’s thermal status, and then realizing the heating, ventilation, and air conditioning (HVAC) system to meet the demand for thermal comfort and energy savings [2].

Fanger established the classical thermal comfort theory [3] in the 1970s, and researchers have since explored numerous methods for the measurement of human thermal comfort. Traditional measurement methods are divided into three categories: questionnaires, environmental measurements, and physiological measurements. The questionnaire survey method is overly reliant on personnel cooperation and has poor operability; the environmental measurement method uses environmental sensors to detect parameters such as room temperature, humidity, and air flow rate to determine the indoor environmental conditions. Although it has some operability, the sensing device’s location is fixed, and it cannot track personnel positions in real time to meet individual thermal comfort needs. As a result, physiological measurement methods are being researched further.

By measuring various physiological signal parameters and associated body responses with sensing devices, physiological measurements are used to determine the body’s thermal sensations. It has been discovered that heart rate, pulse, blood perfusion, skin temperature, body metabolic rate, electroencephalogram (EEG), surface electromyography (sEMG), and other physiological signals can all be used to calculate human thermal comfort [4,5,6,7]. Thermal comfort can also be predicted by changes in body posture and gestures [8]. Physiological measurement methods are classified into three types based on whether they come into contact with the human body: contact measurements, semi-contact measurements, and non-contact measurements. Personnel must wear instrumentation or wearable sensing devices for the first two types of methods. The device installation position and angle, and the personnel’s foreign body sensations, can all cause experimental errors, making it difficult to use in practice.

#### 1.1.2. Outdoor Environment Monitoring

The problem of urban overheating has been exacerbated by rapid global urbanization and global warming [9]. The urban heat island effect and heatwave disasters have deteriorated urban habitats, significantly reduced the thermal comfort of urban residents, and seriously threatened the population’s physical and mental health, as well as economic development. It is critical to create a pleasant outdoor environment, improve urban livability, and boost urban vitality [10,11].

Outdoor thermal comfort is the result of a complex interaction between the urban microclimate (air temperature, humidity, wind speed, solar radiation, and so on) and individual physiological (age, gender, physiological activity, and so on) and psychological factors. Multiple aspects of the natural and artificial urban environment influence pedestrian comfort under the stimulation of multiple senses, such as thermal sensations, vision, hearing, air quality [12,13], and the activities of others [14,15,16]. In the age of big data, the proliferation of navigation and positioning devices, mobile devices, and mapping services has resulted in new types of image geodata. Images depict the urban physical space from various perspectives, thereby assisting quantitative studies of the urban environment.

#### 1.1.3. HVAC Equipment Monitoring

Fault detection and diagnosis (FDD) technology first appeared in HVAC and building engineering in the late 1980s. The majority of the research focuses on the core equipment and piping of the refrigeration plant room. The core equipment includes chillers, chilled water pumps, cooling water pumps, water collectors, collectors, and heat pumps. The piping includes chilled water circuits, cooling water circuits, etc. Typically, FDD technology detects and diagnoses common faults by measuring the temperature or pressure and thermodynamic relationships at various locations in the system, which can effectively extend the lifetimes of equipment and components, stabilize room temperatures, and improve the building’s energy efficiency. Traditional FDD methods in HVAC fall into three broad categories: quantitative model-based methods, qualitative model-based methods, and process history-based methods. Quantitative models are developed based on good physical or engineering principles, but the calculations are more complicated; qualitative models are easy to develop and apply, but they rely on the expertise of developers, and certain rules may not apply when the system is complex. Process history models are a type of black-box model built when one is unfamiliar with the physical characteristics of the system, although the development process is not difficult to implement.

In the traditional sense, HVAC system operational data are structured data from the set of building automation systems (BASs). The use of contact measurement methods such as temperature and humidity sensors to obtain operating data such as the temperature and humidity of equipment or ducts is simple, but it is difficult to avoid impacting the normal operation of the system and creating errors in the measurement data, which affect the fault diagnosis results. As a result, the development of a non-invasive measurement method based on image signals to diagnose and solve faults quickly and accurately would be a breakthrough for FDD in the HVAC field.

### 1.2. Article’s Contributions

Based on the research background presented above, it is known that, in recent years, AI fields such as CV, machine learning, IoT, and robotics have been developed and their applications have been expanded. Non-contact measurements using CV technology and AI algorithms have seen significant advances in three areas: indoor environmental monitoring, outdoor environmental monitoring, and HVAC equipment monitoring. In this review, relevant work in these three fields over the past few years is comprehensively summarized; the main applications of non-contact measurement based on CV technology are presented; and an outlook on the challenges faced in its development, as well as its future development, is elaborated in order to offer suggestions for valuable future research directions.

**Figure 1:**
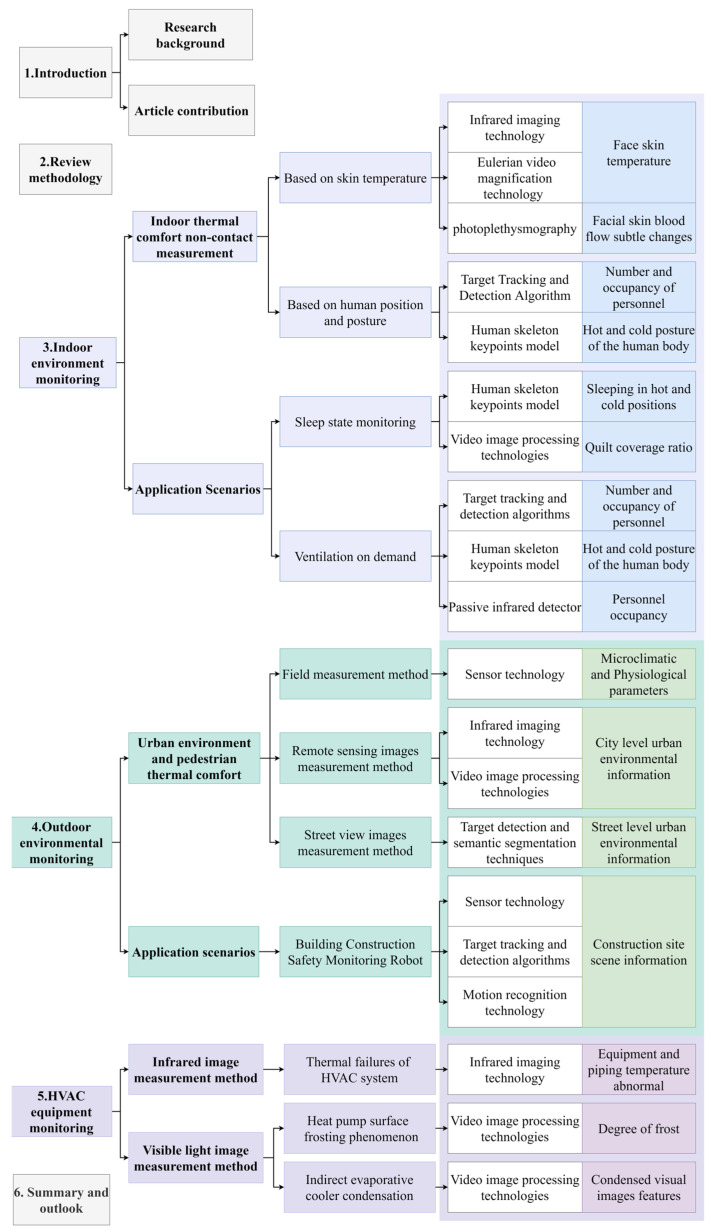
The logical framework for the research.

To achieve these goals, this paper presents a new organizational framework as follows. First, the research background is outlined. Second, the methodology of the review and pertinent materials are summarized. The third section discusses the field of indoor environmental monitoring, the pertinent techniques for non-contact measurement, and their application in two scenarios: sleep state and on-demand ventilation. The following section describes the research conducted on the application of CV techniques and machine learning algorithms to outdoor environmental monitoring. In addition, CV techniques are combined with robotic automation technologies, with an emphasis on their application to building construction safety. Section 5 describes the condition monitoring of HVAC equipment using CV techniques. To illustrate the precision of non-contact visual intelligent monitoring, the research content of CV technology based on visible images applied to two phenomena, heat pump frost and heat exchanger condensation, is described. The conclusions of this paper address the use of new technologies in indoor environmental monitoring, outdoor environmental monitoring, and HVAC equipment monitoring, as well as their possible combinations and research opportunities. The logical framework for the research presented in this paper is shown in Figure 1.

## 2. Review Methodology

In this study, a content-analysis-based literature review methodology was used. The specific process of the literature search and selection was as follows.

### 2.1. Literature Search

The literature research was conducted using Google Scholar, Web of Science, Science Direct, and keywords (Table 1). To ensure the relevance and high quality of the literature, the keywords and Boolean operators “AND” and “OR” were used together to conduct a comprehensive search for publications related to the fields of indoor environmental monitoring, outdoor environmental monitoring, and HVAC equipment monitoring based on computer vision technology. In addition, the references in the search results were scrutinized. Although the percentage was relatively small, strongly relevant literature with milestones appearing in the references was included in the review, even if it did not fall within the search time and English language limits; see Table 1.

### 2.2. Selection Criteria

**Figure 2:**
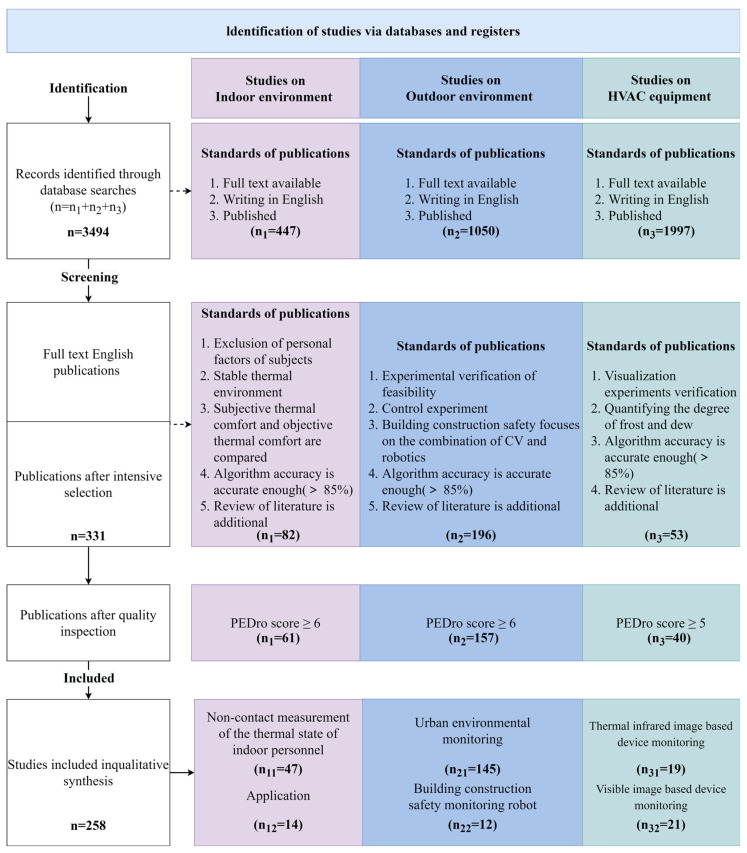
Flow chart of the literature search and selection process.

Figure 2 depicts the screening and adoption of the literature. The procedure was as follows. The literature was screened using the following criteria after excluding non-English, incomplete, or unpublished material:

- Considering the influence of individual factors such as the subjects’ physical health status, gender, age differences, and so on;
- Ensuring the stability of the thermal environment in which the subjects are located during the experiment;
- Combining sensing equipment and CV technology to adequately capture and compare climate parameters, subjective evaluations of subjects’ comfort, and objective physiological parameters;
- Simultaneous acquisition of frost dew visual characteristics and equipment operating parameters using a visualization lab bench based on cameras and sensing equipment to compare experimental results;
- During the experiment, the effect of environmental factors such as light and angle on the frost dew image of the equipment can be weakened by the use of fill lights;
- The level of condensation on the equipment can be lowered by using fill lights.

The literature was screened, and its quality was determined using the Physiotherapy Evidence Database (PEDro) scales. Each article in PEDro corresponds to the subject criteria, and each article is worth one point. As shown in Figure 2, each of the three fields was counted separately, yielding a score of 7, 7, and 6, respectively. As a result, the scores of 6, 6, and 5 were chosen as critical points by three reviewers. The review included literature with scores that exceeded the thresholds. Following further discussion, controversial literature was highlighted.

## 3. Indoor Environment Monitoring

To monitor a person’s thermal state, a non-contact measurement method based on CV technology can effectively compensate for the shortcomings of traditional measurement methods and achieve personalized, high-precision, real-time non-contact measurements. The majority of current non-contact measurements are based on skin temperature, human posture, and personnel occupancy.

### 3.1. Non-Contact Measurement of the Thermal State of Indoor Personnel

#### 3.1.1. Based on Skin Temperature

Skin temperature is closely related to a person’s thermal state and is an effective indicator for the objective evaluation of a person’s thermal sensations and thermal state [17]. Non-contact skin temperature measurement is currently possible using thermal and visible images acquired by thermal infrared cameras and optical cameras, respectively, and image analysis using infrared imaging technology and optical technology, respectively.

- Infrared thermal-image-based skin temperature measurement.

Infrared imaging technology is a non-contact temperature measurement technology that uses thermal images. Thermal infrared (TIR) cameras can directly measure the skin temperature of exposed areas such as the face and hands to monitor personnel’s thermal status and provide a foundation for HVAC system regulation [18]. Infrared imaging can also be used to assess physiological parameters such as the heart rate, blood perfusion, and respiratory rate [17].

Along with advancements in infrared imaging technology, low-cost TIR cameras that are compact, easy to install, and privacy preserving have emerged [19]. TIR cameras can be used to measure the temperatures of different regions of the face, such as the forehead, nose, ears, and cheeks, and predict thermal sensations [20,21,22]. Initial IR imaging techniques were not very mature and required the artificial delineation of regions of interest (ROI) in thermal images, followed by temperature extraction, which was less tractable [18,23]. Researchers have further developed automated methods [19,24], but, while they can automatically locate faces as ROI regions from thermal images, these methods are heavily influenced by the person’s posture, motion, and facial angle. When a person moves, the face assumes a non-orthogonal angle, resulting in inaccurate ROI region detection.

To improve the accuracy in locating facial ROI regions, researchers have proposed combining infrared imaging techniques, computer vision, and machine learning to predict a person’s thermal status. Aryal et al. [25] used a combination of thermal images captured by a TIR camera and visible light images captured by a regular camera. The detected facial region in the visible image was used to locate the facial skin region in the thermal image and extract the facial skin temperature. Kopaczka et al. [26] combined algorithms for face feature detection, emotion recognition, face frontalization, and analysis to further process the infrared face image. Ghahramani et al. [27] used the same method as Aryal et al. to obtain the entire facial skin temperature. However, because only the absolute temperature is measured, calibration drift is unavoidable when aligning visible and thermal images. The accuracy of the developed thermal comfort prediction model was 65.2%. To avoid the effects of these errors, He et al. [28] measured both facial and hand temperatures and predicted the person’s thermal state using a random forest model. It was discovered that the temperature variables were, in order of importance, the cheek temperature, hand temperature, and nose temperature. The absolute temperature or the temperature difference between the different parts of the body can be used to predict the thermal states of personnel more accurately by combining cheek and hand temperature statistics with the nose temperature. After two independent field studies and laboratory research data validation, the model prediction accuracy was found to be approximately 70%, and the model had some adaptability and validity.

To overcome the effects of a person’s posture, movement, and angle, some researchers have considered combining thermal images captured by a TIR camera and red–green–blue–depth (RGB-D) images captured by a Kinect depth camera [29,30]. Cosma et al. [31] used RGB-D data to identify different body parts (head, torso, hands, shoulders, etc.) and combined thermal images to extract skin and clothing temperatures; they then analyzed the data using four machine learning algorithms: support vector machine (SVM), a Gaussian process classifier (GPC), a K-nearest-neighbor classifier (KNN), and a random forest classifier (RFC) [32]. According to the research, the difference in skin and clothing temperatures can be used to predict thermal sensations. However, the high-cost TIR cameras and Kinect cameras used in some studies raise the cost of the equipment and reduce the method’s scalability. In fact, it would be more convenient, efficient, and cost-effective to acquire human thermal physiological signals directly from visible light images captured by optical cameras.

- Skin temperature measurement based on visible light images.

Optical techniques based on visible images have gradually been applied to non-contact human skin temperature measurement in recent years. Methods such as photoplethysmography (PPG) and Eulerian video magnification (EVM) are common.

PPG is a low-cost non-contact optical technique for the measurement of subtle variations in blood flow [33]. Jung et al. [34] proposed a method to infer a person’s thermal status based on subtle changes in the skin PPG signal amplitude extracted from facial images. To reduce interference with the PPG signal, the method combines independent component analysis and adaptive filtering into a single framework. A positive correlation between the skin temperature and thermal sensation was obtained after conducting experiments on 15 subjects, and the validity of thermal sensation analysis based on visible images was confirmed.

**Figure 3:**
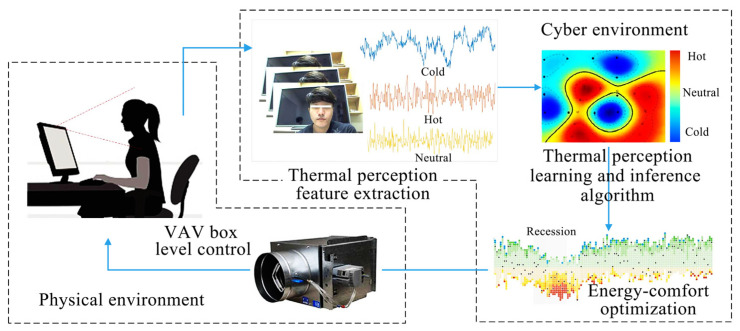
Integrated system of Euler video amplification technology and air supply end conditioning device [38].

EVM is a technique for visual micro-variation magnification [35]. It has been widely used in both structural inspection and medical fields as a CV technique to observe subtle changes in ROI regions in visible images [36]. The EVM technique was first applied in the field of thermal comfort measurement by Jazizadeh et al. [37]. Jazizadeh designed a framework for the identification of a person’s thermal state using the human thermoregulatory mechanism and the EVM algorithm [38]. Subjects working in front of a computer in the experiment were subjected to thermal stimuli at 20 °C and 30 °C, as shown in Figure 3. The camera captured facial images, which were then processed by the EVM algorithm to detect subtle changes in blood flow and identify the state of regulation of the body temperature and the thermal comfort of the human body, which was then fed back to the HVAC system for automated regulation.

**Figure 4:**
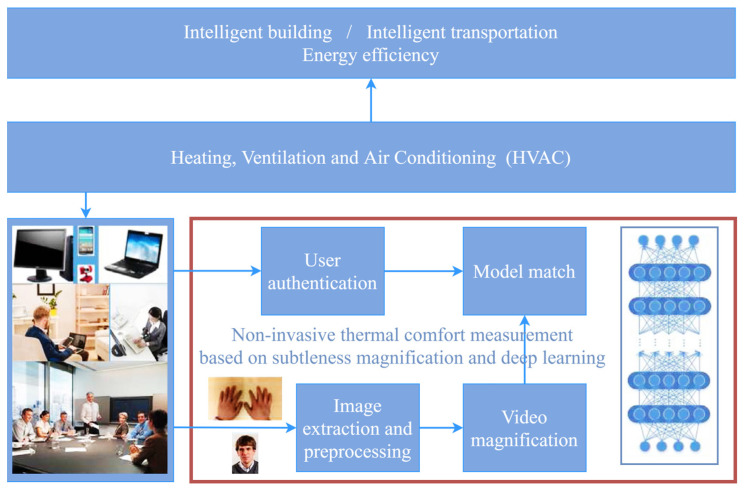
Non-contact thermal comfort measurement in practice [40].

**Figure 5:**
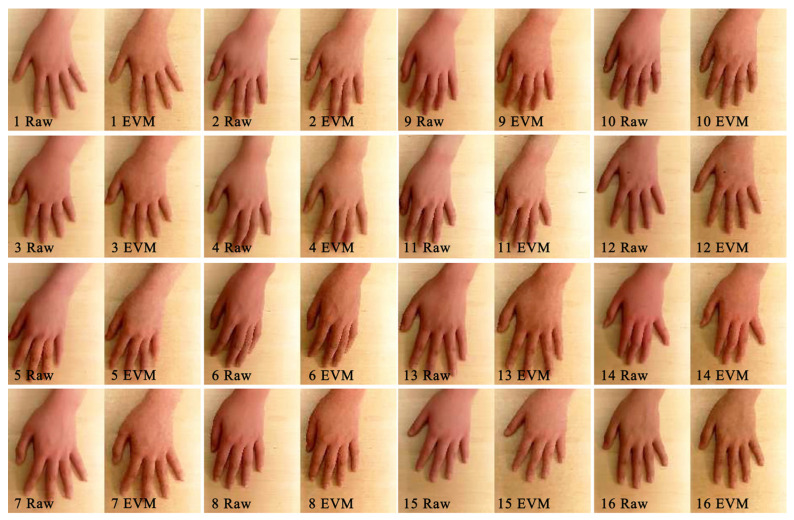
Hand images processed by EVM algorithm [40].

Changes in skin temperature, according to the body’s thermoregulation mechanism, cause blood vessels to dilate/contract, and the skin color then undergoes subtle changes that are imperceptible to the naked eye. Such subtle changes become visible after image magnification by the EVM algorithm. As a result, Cheng et al. [39] proposed a CV-based non-contact human skin temperature measurement technique (Figure 4). They chose young East Asian women as subjects for hand thermal stimulation experiments, and hand images were collected after the hands were stimulated with warm water at 45 °C for 10 min. The EVM algorithm was used to analyze skin color saturation (Figure 5), and a linear relationship between skin color saturation and skin temperature was established using a deep convolutional neural network (DCNN), which predicted thermal comfort. The experimental results demonstrated the validity of the developed individual saturation–temperature (ST) model, with median absolute errors ranging from −0.10 °C to 0.06 °C. Cheng et al. [40] combined EVM and deep learning to create a subtleness magnification and deep learning (NIDL) model and a partly personalized ST model (NIPST). Using 1.44 million sets of hand skin feature data as the dataset, the accuracy of the NIDL model applied to non-contact measurements of Asian females was validated with a mean error of 0.476 °C and a median error of 0.343 °C. However, the preceding study did not take into account individual differences, and, to address this, Cheng et al. [41] proposed a non-contact skin temperature measurement method based on the skin sensitivity index (SSI) and deep learning. The significance of SSI in this deep learning framework was validated using the above hand image dataset, demonstrating that SSI is an excellent high-weight parameter.

#### 3.1.2. Based on Human Position Posture

**Figure 6:**
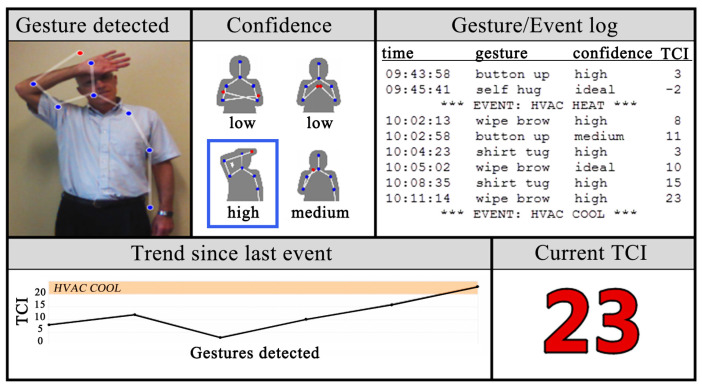
Non-contact thermal comfort measurement in practice [54].

When people are thermally uncomfortable, they often adopt unusual postures or movements, according to research. Human position and posture information can be used to forecast thermal comfort. Human posture recognition technology, whether for single [42,43,44] or multi-person [45,46] recognition, has advanced significantly in recent years, with applications in somatic games, healthcare, and other fields [47,48]. The human skeleton keypoints model [49] is a deep neural network (DNN)-based algorithm that can recognize a moving human body and capture person localization information from a distance. The OpenPose algorithm was used by Cao et al. [50] to advance the field of multi-person location pose recognition [51]. To improve the recognition accuracy, OpenPose learns image features and image-related spatial models. Deepercut [52,53] and other position pose estimation methods strongly support the methodology of the non-contact measurement of a person’s thermal state based on their position pose. Microsoft’s Kinect, a 3D body sensing camera, incorporates functions such as motion capture and image recognition. Meier [54] used Kinect to capture and define four thermally relevant postures, as well as to calculate the corresponding thermal comfort index (TCI), which strongly validated the relationship between human posture and thermal comfort (Figure 6). Kinect also has the ability to predict human thermal sensations and metabolic rates. Because Kinect is not open-source and is protected by copyright, the OpenPose algorithm combined with human skeleton keypoints technology is frequently used in research.

**Figure 7:**
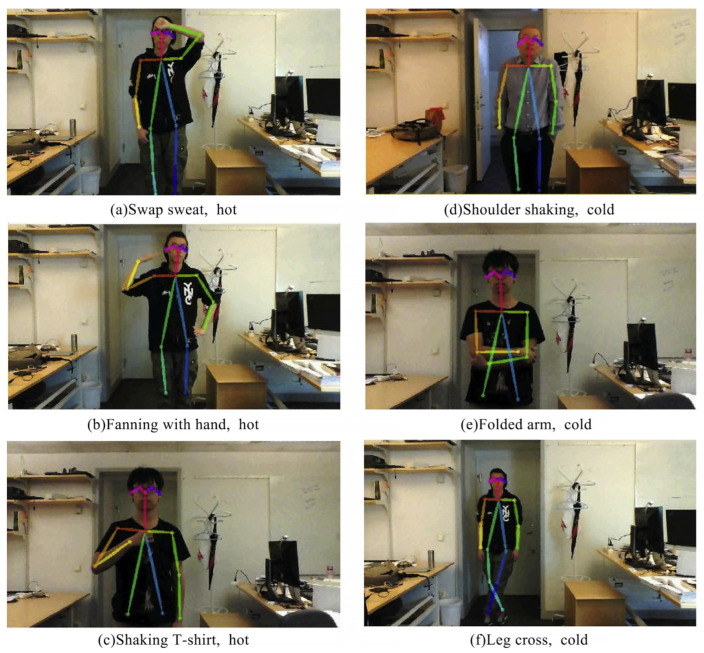
Human pose recognition based on human skeleton keypoints model [58].

Xu et al. [55] extracted thermal adaptation behavioral actions to create a personal thermal demand (PTD) prediction model based on a general camera. Experiments were conducted to demonstrate the accuracy of the proposed model framework for action classification and thermal demand prediction. In a multi-person office setting, accuracy of 91% was achieved. Liu et al. [56] proposed a method to obtain 3D body landmark locations by combining 2D keypoint data from OpenPose and an RGB-D camera. Wang et al. [57] designed and validated an indoor positioning system (CIOPS-RGBD) based on an RGB-D camera. The system employs OpenPose to acquire keypoints of the human body from multiple perspectives, fuse depth data for 3D reconstruction, and predict the person’s position and posture in real time. Experiments show that CIOPS-RGBD adapts well to densely populated complex indoor scenes and improves the indoor environment creation system. Yang et al. [58] proposed a non-contact measurement method that is based on an RGB camera and a human skeleton keypoints model. As shown in Figure 7, 12 thermally uncomfortable postures were defined, and the proposed method’s accuracy in predicting thermal comfort was cross-validated using a questionnaire. Although the human skeleton keypoints technique facilitates the development of individual thermal comfort models, the human skeleton keypoints model has significant technical limitations. In fact, thermally uncomfortable postures can be caused by factors unrelated to cold/heat sensation, and current posture definitions do not yet cover all hot and cold postures, allowing for misclassification.

A passive infrared (PIR) detector is a sensor that detects the infrared radiation that an object emits or reflects [59]. It is well suited for indoor personnel location because it can absorb human infrared radiation that is invisible to the naked eye (human radiation is primarily concentrated in the wavelength range of 9–10 µm) [60,61]. It is now widely used in the surveillance field due to its small size, low power consumption, high sensitivity, low price, and wide detection range.

The PIR sensor can send the personnel location information as a feedback signal to the HVAC system’s control unit, which can then control the background air conditioning system. The air conditioning system adjusts the operation mode based on the person’s occupancy, saving a lot of energy. PIR sensors are frequently used in conjunction with other technologies because they cannot recognize stationary human bodies. Other types of sensors and PIR sensors are usually responsible for separate operational tasks and must be triggered at the same time in order to activate the control signal [62]. According to studies, the use of this technology can increase energy savings by up to 30% [63].

### 3.2. Application

#### 3.2.1. Initial Exploration of Sleep State Monitoring

Sleep quality is important for human health and can be influenced by a number of factors such as health, mood, and sleep environment. Numerous studies have demonstrated that the indoor thermal environment has a significant influence on sleep quality.

There are two types of sleep state monitoring methods available today: contact and non-contact. Traditional contact measurement techniques have numerous flaws. The questionnaire method, which relies on the subject’s sleep memory to assess the previous day’s sleep, is much less reliable and accurate; wristband sleep monitors cannot obtain information about sleepers’ sleep cycle distribution; and polysomnographs require instrumentation components that interfere with normal sleep, causing the “first-night effect” and reducing the measurement accuracy. Non-contact methods are further classified as auditory-based and visual-based. The auditory-based method is not very practical because it has strict requirements for the quietness of the sleep environment. As a result, the visual-based method for the detection of the thermal comfort of human sleep has received a lot of attention.

The vision-based method for sleep thermal comfort detection is advantageous for the collection of more sleep-related data. Many movements occur during sleep, such as eye movements, rolling over, jaw movements, leg tremors, eye movements, and so on. These movements become sleep information and are used in the medical community to assess sleep quality. Researchers collect sleep image/video data and extract sleep-related information using the Eulerian video amplification technique, the human skeleton keypoints model, and machine learning algorithms to determine sleep quality. Peng et al. [64] proposed a multimodal sensing system that integrates visible images, thermal images, and heart rate signals. The system classifies the multimodal signals using a support vector machine (SVM) and fuses the multimodal outputs together to infer the sleep state. The results show that the proposed system is successful in distinguishing between sleep and waking states. Choe et al. [65] created an automatic videosomnography (VSG) method that models the relationship between human head movements and sleep states using machine learning. A number of non-contact posture measurement methods that are highly instructive for sleep state monitoring have emerged in recent years. Mohammadi et al. [66] used a TIR camera in conjunction with a deep learning algorithm to measure sleep posture automatically. The TIR camera was used to capture realistic sleep thermal images of 12 subjects in a thin blanket situation, which were then fed into a deep learning network to classify the four delineated sleep postures. According to the experimental results, ResNet 152 had the highest classification accuracy of more than 95% among the seven deep networks tested. Piriyajitakonkij et al. [67] developed an ultra-wideband (UWB) radar-based method for the detection of sleep states. To improve the detection accuracy, the research process employs deep learning algorithms to classify the sleep pose and fuses the time-domain–frequency-domain signals via the multi-view learning (MLV) method. Despite their increasing computational complexity, these methods do not significantly improve the accuracy of sleep pose detection.

**Figure 8:**
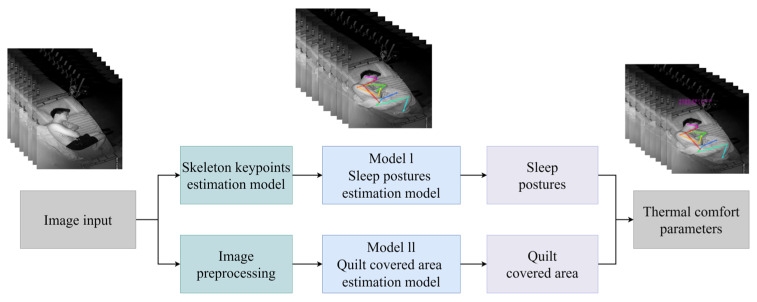
New vision-based non-contact human sleep thermal comfort detection.

Cheng et al. [68] proposed a novel vision-based non-contact method for the detection of human sleep thermal comfort. Based on 438 valid questionnaires, the method defined 10 thermal comfort sleep postures, as shown in Figure 8. The thermal comfort sleep posture dataset was created by collecting data from 2.65 million frames of sleep postures in their natural sleep state. The basic framework and model of human sleep posture detection algorithm were constructed using the residual idea and long short-term memory (LSTM) algorithm based on the large amount of data collected. The human skeleton keypoints technique was used to determine the person’s sleep posture, and the video image processing technique was used to obtain the quilt coverage. The results showed that the proposed sleep thermal comfort detection method had average accuracy of 91.15%, as well as obvious robustness and effectiveness.

In order to monitor more types of sleep action postures in the future, the algorithm and the detection performance must be improved. In the future, the intelligent regulation of the sleep thermal environment will be studied further, and the HVAC system parameters will be automatically adjusted in real time to avoid overcooling/overheating supply and meet the demand for human sleep thermal comfort. The non-contact sleep thermal comfort monitoring method will be valued and applied in the field of elderly care as China’s population ages.

#### 3.2.2. Ventilation on Demand

**Figure 9:**
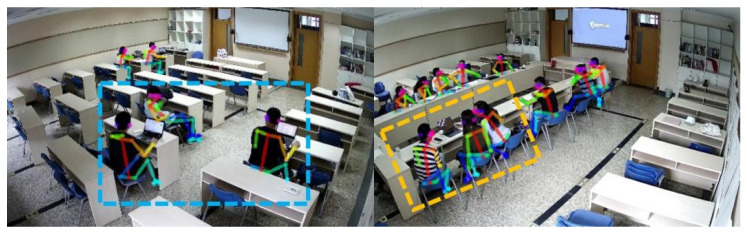
Non-contact measurement in a multi-purpose lecture hall [69].

On-demand ventilation can be achieved in an energy-saving ventilation strategy via the non-contact measurement of personnel position postures to ensure energy savings and comfort. Wang et al. [69] proposed a personnel positioning system based on a human skeleton keypoints model, as shown in Figure 9. This system detects room operation patterns in a multi-purpose lecture hall (classroom/meeting room) by identifying and estimating personnel occupancy and regulates the HVAC system accordingly. Experimental results show that in small and medium-sized indoor spaces, the system can complete image acquisition, extraction, 3D reconstruction, and data fusion in 1.5 s, as well as performing real-time human positioning and pose recognition. The environment of large open indoor spaces, on the other hand, is more complex, and the tracking and positioning of indoor occupants, as well as the real-time regulation of air conditioning systems, face new challenges. Cui et al. [70] proposed an intelligent zonal ventilation control strategy based on people’s occupancy situation based on this. AS-DA-Net is used in the strategy to identify the number of heads in video images, predict the occupancy density of each partition, detect occupancy dynamically, and automatically regulate the air supply volume of each partition. The experimental results show that the proposed scheme for large open indoor spaces is effective. It considers the balance of personnel thermal comfort, indoor air quality, and energy consumption, in comparison to the existing CO_2_ concentration-based ventilation control.

**Figure 10:**
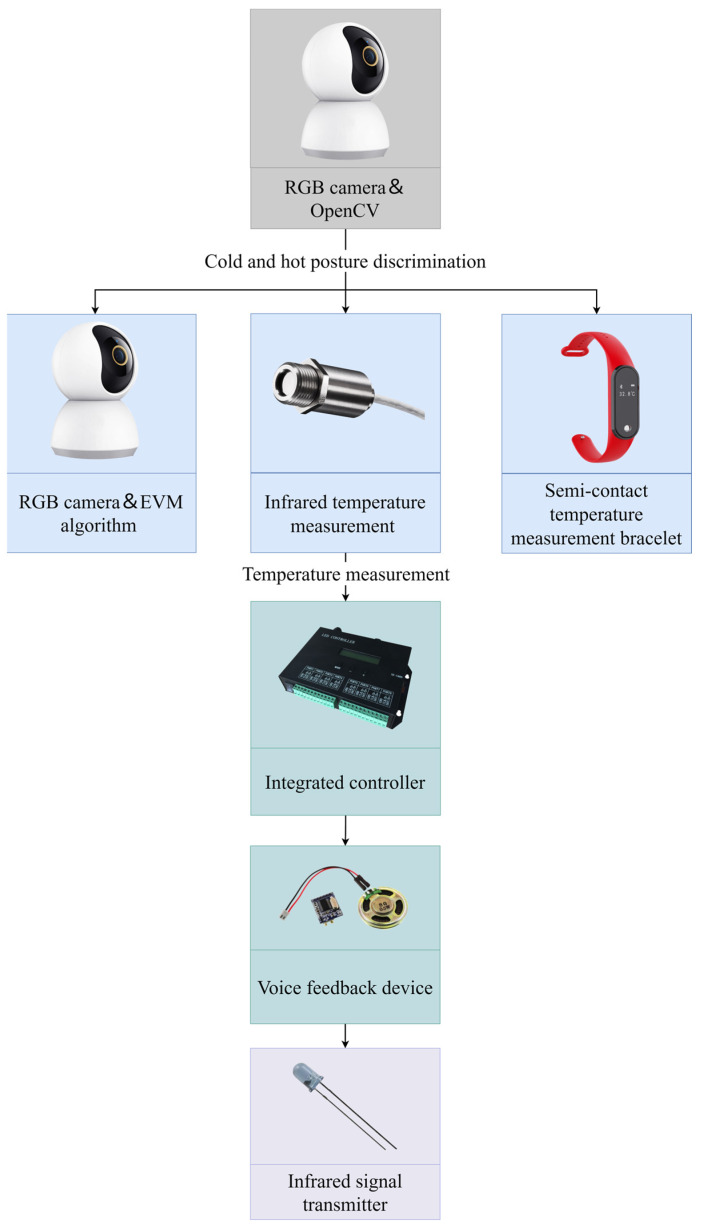
Non-contact automation control process of micro-environment air supply device.

It is also possible to change the air direction, air speed, and air volume parameters of the air conditioning system in real time to meet the cooling/heating needs of personnel by monitoring their thermal sensations in the human-centered ventilation strategy. Figure 10 depicts a micro-environmental air supply device with non-contact automation control. The subject working in front of the computer will receive three types of temperature measurements at the same time during the experiment. The RGB camera detects the subject’s thermal discomfort or cold discomfort posture while also capturing the subject’s facial image and applying the EVM algorithm to calculate the facial temperature; the TIR camera measures the facial temperature; and a semi-contact measurement device, such as a thermometric bracelet, measures the skin temperature. In the integrated development board, the results of the three types of temperatures are compared, and the temperature measurement results are used to predict the subject’s real-time thermal sensations.

However, due to the small exposed area of human skin, the EVM algorithm will produce temperature measurement errors; the infrared temperature measurement technology will produce temperature measurement errors due to a redundant light source or the shaking of the personnel’s body; the long-term wearing of the bracelet will cause the personnel to experience discomfort, and those who cannot wear it continuously will cause missing data. To avoid system errors, a voice feedback device is programmed to ask personnel about their thermal sensations, compare them to the predicted heat sensations, and control the end device based on the result of this check.

The speed of data extraction, analysis, and signal transmission in non-contact measurement based on CV technology is faster than the speed of mechanical equipment (valves, fans, etc.). The mismatch in operational processing speed impedes the practical application of on-demand ventilation technology and non-contact measurement technology. As a result, Zhai et al. [71] proposed combining an energy-efficient fan with a higher air conditioning background temperature without changing the room setpoint. The energy-saving fan’s adjustment speed corresponds to the processing speed of the non-contact measurement technique, avoiding the limitation of the air conditioning system’s slow adjustment speed. However, the room’s size, its irregular shape, and the mutual occlusion of people can all lead to CV technique misjudgments.

The rational use of natural ventilation in an on-demand ventilation strategy can balance energy efficiency and thermal comfort needs. The degree of window opening affects the efficiency of natural ventilation. There are two types of methods for the monitoring of window status based on common cameras: traditional and novel. Traditional window condition monitoring relies on image processing techniques to extract the intensity of the image pixel distribution. Bourikas et al. [72] proposed and validated a camera-based method for the evaluation of the window opening type and degree based on window pixel intensity distribution images. The measurement’s precision was greater than 90%. However, in practice, the fixed location of the camera would limit the number of façade monitoring stations and thus the sample data. Zheng et al. [73] calculated the percentage of window opening based on the intensity distribution of window pixels. To obtain a larger sample, the experimental procedure was carried out using large-scale data sampling. The sliding windows of a hospital in Nanjing were studied with a recognition error of approximately 8%. Based on CV techniques, Luong et al. [74] developed a method for the monitoring of the condition of building façade windows. The method uses image segmentation techniques to automatically segment the individual images of each window with accuracy of 89%. The limitation is that it is only applicable to shaded windows, and the manually adjusted window status thresholds are not scalable. In fact, window monitoring methods based on the pixel intensity distribution are easily influenced by light levels and weather conditions. As a result, deep learning algorithms have been introduced into the field as novel window state monitoring methods. Tien et al. [75] developed and validated a deep learning method capable of automatically identifying individual window states in real time. The method’s accuracy was 97.29%, demonstrating the advantages of deep learning for automatic window state recognition. Sun et al. [76] proposed a method for the automation of the real-time monitoring of window states in severe cold regions, combining CNNs and image processing techniques. Experiments showed that in the transition season, severe cold regions prefer large window opening angles, and the window opening probability in the southeast direction is greater than in other directions. The method is highly scalable and can be combined with building energy consumption and other factors to facilitate the analysis of multiple application scenarios. Window monitoring using TIR cameras can overcome the limitations of optical cameras and is especially useful in observing window states at night. Chen et al. [77] created a remote sensing method based on TIR cameras to identify indoor temperatures. The results demonstrated that full-opening window IR images could clearly quantify the indoor temperature in the heating, excessive, and cooling states. The absolute deviation between the measured infrared temperature and the true value of the temperature at different heights was 0.5 °C for the heating and excessive states, and the deviation was greatest for the cooling state. Future research could look into the differences between daytime and nighttime window opening patterns during the transitional season.

## 4. Outdoor Environment Monitoring

### 4.1. Urban Environmental Monitoring

**Figure 11:**
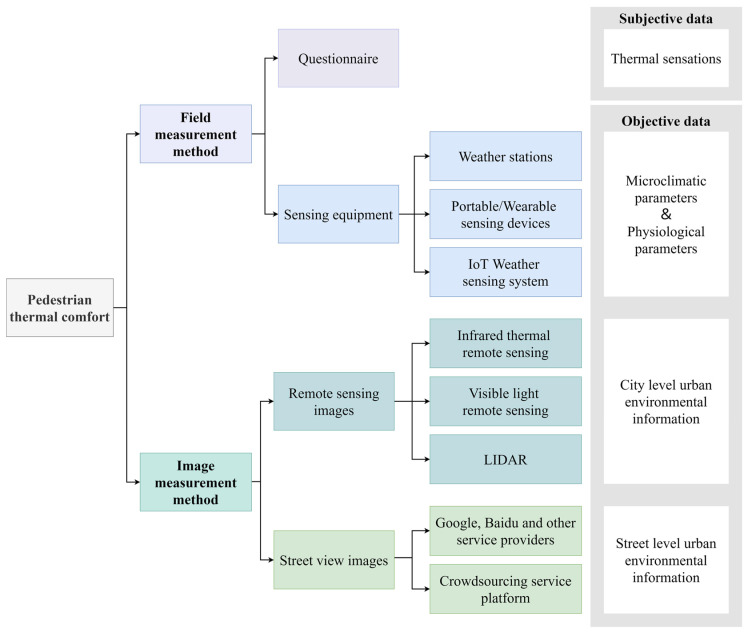
Methodological framework for collection of different types of data to measure pedestrian thermal comfort.

Urban environmental monitoring requires the collection of both subjective human perception and objective environmental data. There are two categories based on the data monitoring methods: field measurement methods and image measurement methods (Figure 11).

#### 4.1.1. Field Measurements

Traditional outdoor thermal comfort measurement is a field survey method based on questionnaires and outdoor parameter measurements. The subjective perceptions of subjects are obtained through questionnaires, and environmental and physiological parameters measured by sensing devices are used to obtain thermal comfort evaluation indices such as PET, PMV, UTCI, SET*, and so on [78], which are mapped to human thermal sensations for outdoor thermal comfort modeling. The questionnaire method is simple and straightforward, but it can interfere with pedestrian behavior patterns. The microclimatic environment during the survey can change, which can affect the accuracy of the results [79]. The outdoor parameter measurement method is primarily based on a network of weather stations and various sensing devices to obtain microclimatic parameters such as the temperature, relative humidity, barometric pressure, wind speed and direction, solar irradiance, and rainfall [80,81,82]. The microclimatic conditions, particularly temperature, have a large impact on outdoor thermal comfort [83,84]. There are two types of weather stations: stationary and mobile [85]. Although fixed weather stations allow long-term observations of meteorological data [86,87], the amount of information can be limited by the location and number of weather stations, making it difficult to adequately display spatial variations in heat, and the equipment maintenance costs are high [88]. Mobile weather stations compensate for the fixed type by installing sensing equipment in a vehicle at a height of 1.5 m from the ground (the average height of the human heart) and collecting data in a mobile manner based on a dedicated vehicle [89,90]. However, the method is still constrained by the application scenario.

**Figure 12:**
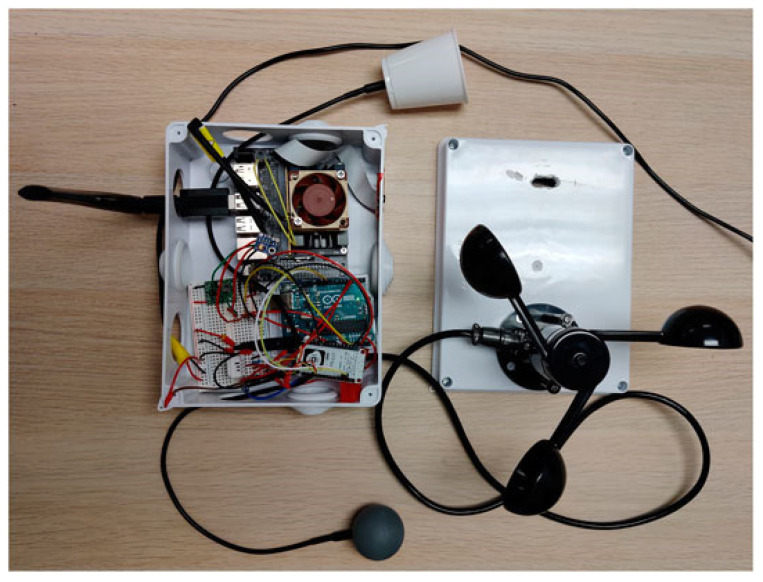
Top view of the MaRTiny device. Jetson Nano with cooling fan, camera, and WiFi module. Arduino board connected to different weather sensors [99].

To overcome the limitations of mobile measurements in vehicles, portable/wearable sensing devices with strong communication capabilities and low costs are gaining popularity [91]. Wearable devices that integrate miniature weather stations and embedded sensors with helmets and backpacks can continuously monitor spatial and temporal changes in information such as microclimatic parameters [92,93], physiological parameters [94], geographic locations [95], concentrations of various pollutants [96,97], and noise [98] in real time, and they transmit and store the data via wireless networks. On this basis, Kulkarni et al. [99] integrated vision systems and machine learning algorithms into an Internet of Things (IoT) weather sensing system and proposed MaRTiny, a new low-cost computer vision biometeorological sensing device. As shown in Figure 12, the meteorological system of this device can passively collect microclimatic data and estimate the mean radiation temperature (MRT) using the SVM algorithm. The vision system employs pedestrian detection (YOLOv3) and shadow detection algorithm (BDRAR network) models based on the NVIDIA Jetson Nano development board, counts the number of people in the shade and sunlight using the camera, and uploads the data to Amazon Web Services (AWS) servers. The study’s findings show that the root mean square error (RMSE) of the MRT estimated based on machine learning is reduced from 10 °C to 4 °C in the meteorological system, and the accuracy of pedestrian detection is 95% and that of shade detection is 80% in the vision system. The observations of the meteorological system and the vision system are consistent. The data collected by the MaRTiny device can effectively analyze the impact of the urban microclimatic conditions on people’s behavioral patterns in public spaces (e.g., the number of people holding umbrellas and taking transportation) to guide the management and design of urban greenery and improve urban thermal comfort.

Field measurement methods have long data collection update cycles, are limited in the urban areas that they can cover, are time-consuming and labor-intensive, and are not appropriate for large-scale applications. As a result, building IoT systems in the field of urban environmental monitoring with low-cost and integrated sensor technologies and artificial intelligence has become an important means of shifting from traditional to new types of observation.

#### 4.1.2. Remote Sensing Image Measurement

Remote sensing images are those obtained by photographing or scanning the Earth’s surface with remote sensors installed on remote sensing platforms. The remote sensing images are processed or recoded to produce remote sensing images that can be used as the foundation for outdoor thermal environment and thermal comfort studies. Remote sensing technology is classified into infrared thermal remote sensing, visible light remote sensing, LiDAR, multispectral remote sensing, and so on based on the electromagnetic wave spectral band range.

**Figure 13:**
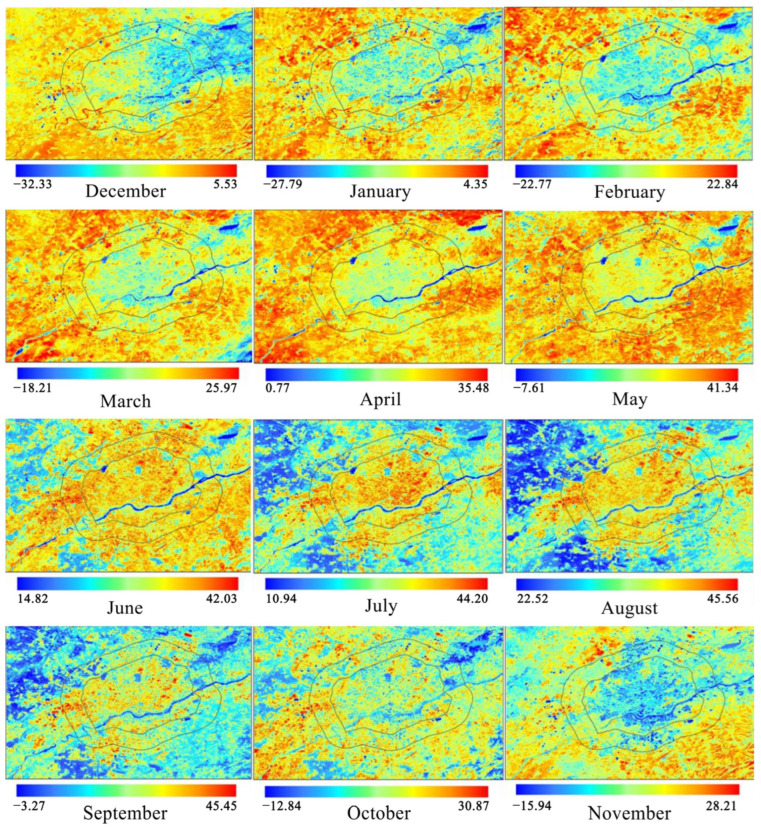
Thermal image of ground surface temperature (LST) in Shenyang, China, in 2020 [101].

Satellites are primarily used in infrared thermal remote sensing technology to acquire thermal images and calculate the land surface temperature (LST) (Figure 13) [100,101]. Satellites such as Landsat and NOAA were successfully launched in the 1970s, and they began to provide data support for urban observation, climate and environment research, and other scientific research fields. Thermal images were used by researchers to measure LST and initially explore the urban thermal environment [102]. The combination of thermal images and measured meteorological data is commonly used in the assessment of urban heat islands [103,104], urban heat flux [105,106], urban parameters and other urban scale issues, and the thermal environment. An advanced, very-high-resolution radiometer (AVHRR) is installed on NOAA satellites. Stathopoulou et al. [107] proposed a method for the estimation of the discomfort index (DI) from thermal images based on this. When compared to the DI values calculated from meteorological data, it was discovered that the DI values could be effectively estimated using thermal images with a resolution of 1.1 km to measure human thermal sensations. In terms of spatial details, Xu et al. [108] improved this estimation method. It was demonstrated that high-precision DI images with a resolution of 10 m could distinguish three types of thermal discomfort, reflecting spatial differences in the urban environment in terms of building, vegetation, and water content. Mijani et al. [109] used Tehran, Iran, as their study site to propose and validate a least squares method (LSM) for outdoor thermal comfort modeling based on thermal remote sensing images and climate data. As inputs, the model takes urban environmental parameters and human physiological parameters, and it outputs DI values. The correlation coefficient between the true and predicted DI values exceeds 0.85. Although thermal remote sensing images can provide urban-scale LST, the data are constrained by the acquisition time of the satellite. Furthermore, the surface temperature derived from remote sensing data represents the temperature of the top of the tree canopy, building roofs, and the ground, which cannot fully represent the actual thermal stimuli experienced by street pedestrians [108]. As a result, researchers [110,111,112,113] have integrated infrared sensors into devices such as air vehicles, roof viewpoints, ground observation devices, and smartphones to gradually and accurately scale temperature collection from the urban scale to the neighborhood scale, building scale, and microscale [114]. Zhao et al. [115] created a three-dimensional thermal imaging technique. This technique observes and assesses the outdoor spatial thermal environment at the street level by using 2D thermal images acquired by a TIR camera mounted near the ground, in conjunction with 3D models acquired by a UAV to generate 3D thermal images and extract MRT values. Furthermore, a visualization tool integrating thermal images, IoT, and a digital twin platform is being developed to monitor urban environmental data [116].

**Figure 14:**
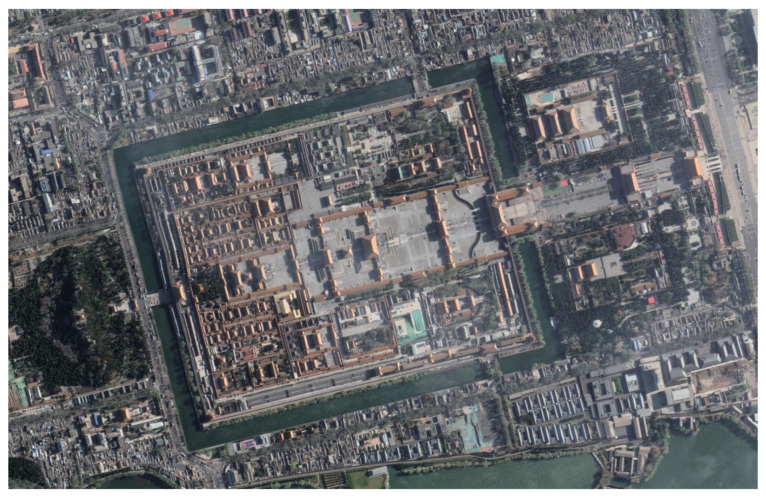
Visible light remote sensing images.

Visible light remote sensing technology allows satellites, aircraft, and other aerial vehicles to observe the ground from above (Figure 14). It is capable of displaying 2D urban features such as roads and bridges, buildings, land use, vegetation greenery, and geomorphology [117,118]. It has more structured and consistent data than traditional images [119,120]. Images of visible light remote sensing are widely used for the analysis, evaluation, and visualization of urban greening and shading levels at macroscopic scales [101,121]. Spatial remote sensing technology has been steadily improving for decades. Combining CV techniques and deep learning algorithms to process visible images can automate image classification [122,123], semantic segmentation [124], and scene parsing [125], resulting in lower labor costs. The green space percentage, green space/building area ratio, tree density, shade coverage, and other indices are commonly used [126,127,128]. Hong et al. [129] used GIS to extract the green space density and pavement from visible light images using deep learning and image processing techniques. Although visible light remote sensing images are macroscopic and fast, they only capture one perspective, lack elevation spatial information, and are unable to capture urban details at the street level. Furthermore, the cost of acquiring high-resolution remote sensing data is high. Multiple data fusion becomes a new analysis method in extracting urban morphology, building information, land use types, and other issues. To analyze urban shading, satellite images and geographic information system (GIS) data can be combined. Hu et al. [130] used LiDAR technology in conjunction with near-ground photography to extract tree canopy lines as a new index to quantify the street tree morphology. The LiDAR technique, on the other hand, is more expensive and unsuitable for large-scale research applications. Radar data decoding and compilation is also time-consuming and tedious. Point cloud data from synthetic aperture radar (SAR), light detection and ranging (LiDAR), multispectral remote sensing, and visible light remote sensing techniques are frequently combined with geospatial data or street view images [131,132,133].

#### 4.1.3. Street View Image Measurement

**Figure 15:**
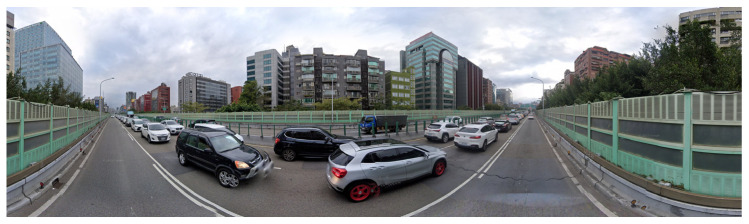
Street view images.

The big data era; the maturity and popularity of navigation and positioning devices, mobile devices, and mapping services; and the rapid development of sensing and digitization technologies have resulted in a new type of geographic big dataset (Figure 15), street view imagery (SVI) [134].

Street view images compensate for satellite images’ deficiencies by recording detailed three-dimensional profiles of city streets from microscopic and pedestrian side view perspectives. Image data provide data support for quantitative studies of the urban physical environment due to their wide coverage, high resolution, large data volume, and low cost. SVI is classified into panoramic images and crowdsourced images, according to data sources. Panoramic images are primarily captured by map service providers who use street view vehicles to traverse the city road network, collecting 360-degree panoramic visual information. Google Street View (GSV), Tencent Street View (TSV), and Baidu Street View (BSV) are all well-known service platforms [135,136]. Mapillary, KartaView, and Apple Map are examples of popular crowdsourcing service platforms. Individual web users capture and provide crowdsourced images. Because of the high quality and perspective of the images captured by users, they are frequently used as a supplement to panoramic images.

Technical support for the quantification of urban physical environments is provided by CV techniques and deep learning algorithms [137]. SVI is used for visual object recognition and the classification of scene types and attributes in physical environment quantification. The primary visual tasks of CV techniques are object detection and object segmentation. The former can identify object position and type information in an image, while the latter can classify each pixel point in an image. DCNN is the most commonly used image analysis model in deep learning, and its representative structural frameworks include AlexNet, GoogLeNet, DenseNet, and others. Deep-learning-based CV techniques can extract multi-level information such as the city geometry, building façade color, city greenery level, and sky view factor (SVF) automatically.

Related research has found that urban geometry and street greenery have an impact on the urban thermal environment and that good urban planning and design can improve outdoor pedestrian thermal comfort [138,139,140,141,142]. It has become popular to study the urban street environment using SVI to quantify the urban geometry and greening level.

- Urban Geometry.

The formation of a street canyon in the center of a city from a cluster of high-density buildings is critical in influencing the urban microclimate and comfort. The height to width ratio, street orientation, and sky view factor (SVF) are the main geometric parameters of urban morphological structures [143]. Oke proposed the concept of the SVF, which is defined as the ratio of the pedestrian-visible sky area at a given surface point to the full-view sky area in the urban street space [144]. This value is a dimensionless number between 0 and 1 that represents the degree of openness of outdoor public spaces and also serves as an index to measure the level of shading in various street canyons [145]. As the morphology of the buildings and street trees on both sides of the street changes, so does the level of sky visibility from a pedestrian viewpoint [146]. A value of 0 indicates that the sky is completely shaded, while a value of 1 indicates that there is no shading at all [147]. Geometric methods [148], global positioning system (GPS) methods [149], simulation methods, and image methods [150] are commonly used to estimate the SVF. The image method is clearly more accurate and straightforward than the other methods [151].

A traditional method for the estimation of the SVF based on fisheye images is the fisheye image method [152]. In a street canyon, a circular fisheye lens is used to shoot squarely up to the sky, projecting the hemispheric environment onto a circular plane and capturing a 2D circular fisheye photograph. To segment the sky area and the occluded area, image processing software is used to perform processes such as binarization, contrast, and brightness adjustment on the fisheye image. The SVF is calculated as a percentage of the visible sky area [153,154]. The fisheye image method accounts for the occlusion of vegetation and other urban infrastructure, resulting in more accurate estimation results. However, the segmentation process in different types of image processing software, such as RayMan and SkyView, relies on manual operation and parameter setting, which is time-consuming and labor-intensive. The fisheye image method, which necessitates field photography, is also affected by lighting and weather conditions, making it unsuitable for large-scale studies [155].

The street-level image method is a low-cost and efficient open-source SVI-based visualization method for the estimation of the SVF [156]. Several researchers [157,158] have generated fisheye images from SVI mapping using hemispherical transformation and then estimated the SVF to evaluate solar radiation and the sunshine duration at the street level. These studies demonstrate the feasibility of estimating the SVF based on SVI, and while effective in reducing the field photography time, the manual image processing time remains lengthy. Several studies have investigated methods of automatically estimating the SVF on a large scale. Xia et al. [159] proposed the DeepLabV3 + semantic segmentation model and a deep-learning-based automatic estimation method for SVF values. The method employs a deep learning model to semantically segment SVI and generate fisheye images in order to compute the SVF automatically. The proposed method recognizes the sky at a rate of 98.62%. To recognize GSV images and estimate the SVF, Liang et al. [160] used an open-source DCNN algorithm called SegNet. Zeng et al. [156] created an SVF estimation toolbox based on SVI, using Python and the OpenCV software library. It can batch-process street view images and estimate the SVF quickly. However, the proposed method’s sky area detection is vulnerable to vegetation occlusion, such as massive tree canopies. Seasonal variations in vegetation can cause errors in SVF estimation. Gong et al. [155] chose the Hong Kong urban area as the study object and proposed a method for the estimation of the sky, tree, and building view factors (SVF, TVF, BVF) of street canyons in a complex urban environment. The method extracts street features from GSV images using the Pyramid Scene Parsing Network (PSPNet) and directly verifies the accuracy of the view factors (VF) estimated using hemispheric photography reference data. Based on this, Gong et al. [161] calculated the solar radiation intensity of street canyons using GSV images and demonstrated the close relationship between the SVF and solar radiation. Du et al. [162] developed a method to obtain the VF from BSV images and automatically estimated the sunshine duration through a similar line of research. Nice et al. [163] proposed an automated system for sky area detection based on an adaptive algorithm to better adapt outdoor images under various weather conditions. A CNN trained on 25,000 images from the Skyfinder dataset adaptively selects the best image processing method (mean-shift segmentation, K-means clustering, and Sobel filters) to improve the detection accuracy. These studies show that the low-cost, automatic, and efficient estimation of the SVF is feasible.

Further quantitative studies of the urban environment using SVI rely primarily on the extraction of data on various urban elements, such as buildings, roads and bridges, and street furniture [164]. Key features extracted by SVI in the building domain, such as the building type and number [165,166], building condition [167,168], year of construction [169,170], and floor height and number of stories [171,172], greatly enrich the building dataset. The color of a building’s façade, for example, has a significant impact on pedestrians’ visual experience and environmental perceptions. Zhong et al. [173] used deep learning algorithms to automatically extract the dominant color of the urban façade (DCUF) from BSV panoramic images. Zhang et al. [174] used a similar approach to add a building function classification module to the scheme while achieving the automatic computational recognition of urban façade colors. These studies serve as a foundation for ideas for urban planning and design, as well as for improving residents’ thermal comfort. CV techniques, deep learning algorithms combined with SVI, POI, satellite remote sensing data, and social media data have also been widely used in recent years for building energy consumption [167], electricity prediction [175], land use [176,177,178], urban functional classification [179,180], road and bridge monitoring [181,182,183], and other areas [184].

- Urban Greening.

Urban greenery is an important part of the urban environment. Green space plants include street trees, bushes, lawns, urban parks, and other types of vegetation. Proper greenery planning and shading design can effectively regulate the urban microclimate, improve the urban thermal environment and pedestrian thermal comfort level [185,186], and improve the visual experience and psychological well-being of urban residents [187,188]. Depending on the measurement method and perspective, there are two major types of urban greenness indices. Traditional urban greenness indices such as green cover [189], the leaf area index (LAI) [190], and the normalized vegetation index (NDVI) [191] are mostly measured and calculated using remote sensing images or GIS from an overhead perspective. Because of the shooting angle, remote sensing images frequently miss shrubs and lawns beneath the canopies of trees, as well as green vegetation on building walls.

Aoki’s Green View Index (GVI) is a horizon-based index that assesses the level of greenery at the street level. The value represents the proportion of green pixels in the pedestrian’s field of view and reflects the degree to which pedestrians perceive their green surroundings. It has been demonstrated that the level of street greenness influences various dimensions, such as house prices [192], finances [193], crime rates [194], and residents’ health [195,196]. The GVI is measured using three methods: field measurement, remote sensing measurement, and SVI measurement. The field measurement method uses image processing software to manually extract green vegetation areas [197]. Street shading and greenery analysis takes time and has a limited number of sampling points, making it only appropriate for small-scale studies. Remote sensing measurements have limited accuracy [198], and remote sensing images and LiDAR data are frequently used in conjunction with SVI data to assess urban greenery [199]. With the advent of the big data era, researchers have used SVI such as GSV images and TSV images as new data sources, combined with CV techniques, to automate the extraction of greening areas and calculate GVI values to compensate for these deficiencies [200,201,202]. Waveband operation, color space conversion, image semantic segmentation, and other commonly used methods for green area extraction have been developed continuously [203]. Dong et al. [204] used Beijing as the study area to extract green vegetation areas in TSV images and calculate GVI values using image segmentation algorithms, demonstrating the feasibility of assessing the greenness of complex streets in megacities using SVI. The GVI calculation process necessitates the extraction of multiple GVI values from various locations in the neighborhood for data aggregation. The choice of aggregation method can affect the final GVI and cause calculation errors. To address these shortcomings, Kuma-koshi et al. [205] proposed an improved standardized GVI (sGVI) index based on Voronoi tessellation to improve the accuracy of street greenness evaluation. Chiang et al. [206] calculated GVI and SVF indices from GSV images and verified the consistency of deep learning and manual classification methods. Zhang et al. [207] extended the application of GVI indices and creatively proposed and validated a method to calculate the optimal GVI path. The visualization of geographic data in complex scenes was realized using the Floyd–Warshall algorithm and Osaka, Japan as an example. All of the preceding studies assess the amount of street greenery based on the GVI index for visualization, analysis, and application.

Researchers have attempted to investigate new greenery assessment indices from various perspectives in order to adequately describe the complexity of the horizontal distribution and vertical structure of street greenery. Tong et al. [208] proposed a new street vegetation structure diversity (VSD) index by combining remote sensing and street view perspectives. The difference in the amount of greenery and green structure between old and new urban areas was demonstrated using Nanjing, China as an example. SVI measurement based on CV techniques and machine learning can automatically acquire street tree features on a large scale [209,210,211]. Liu et al. [212] created an automatic street tree detection and classification model based on SVI and deep learning. To deal with the long-tail effect of street trees, the model accuracy was improved by improving the loss function of YOLOv5. In the GSV depth map, the depth evaluation method was validated for the first time using the deep learning model Monodepth2. The city of Jinan, China was chosen as a test site to obtain the tree species, distribution density, canopy structure, and coverage through visual analysis, and a city-wide tree inventory was established. Yue et al. [213] used the DeepLabv3+ algorithm to efficiently extract shadow areas from panoramic images and proposed and validated a shadow coverage index.

As an important component influencing the urban environment, pedestrian flow data are critical for human-centered urban greening design. Traditional methods of collecting pedestrian flow data include manual counting and cell phone signal counting. It is labor-intensive, has obvious disadvantages, can only be applied to specific study areas, and lacks scalability. The new pedestrian traffic collection method is an image method based on video image processing technology. To overcome the limitation of single surveillance camera coverage, Wong et al. [214] created the OSNet + BDB model, which uses images from multiple surveillance cameras as data sources and can identify pedestrian trajectories and distribution features over a large area. Tokuda et al. [215] used the R-FCN algorithm and the ResNet-101 layer residual network to train a street image dataset and count the number of pedestrians. Li et al. [216] proposed a methodological framework consisting of K-fold max variance semi-supervised learning and DeepLab v3+ (KMSSL-DL). KMSSL-DL combines machine learning and computer vision techniques to estimate the number of pedestrians using unlabeled data from high-dimensional urban features, and it uses the DeepLab v3+ model to identify street trees and plan street trees in a pedestrian-oriented manner. Predictions based on pedestrian analysis are used in scenarios such as train stations [217] and traffic intersections [218].

The proliferation of massive streetscape Images aids in the understanding of urban environments from the perspective of pedestrians, but it also has limitations. On the one hand, streetscape images are not taken at the same time, and the characteristics of the streetscape environment can be affected by different seasons and lighting conditions, resulting in measurement errors. To support the construction of smart cities, the fusion of multiple heterogeneous urban data, such as SVI, remote sensing images, and social media data, is used [219], which has higher requirements for the new generation of information technology represented by artificial intelligence (AI), Internet of Things (IoT), and digital twin (DT) [220,221,222].

### 4.2. Building Construction Safety Monitoring Robot

The occurrence of construction site injuries is extremely high, emphasizing the importance of construction safety management and monitoring. The most common causes of accidents can be divided into two categories: those caused by workers themselves, such as the improper use of personal protective equipment (PPE) or unsafe behavior caused by fatigue, and those caused by the role of workers and the surrounding environment, such as equipment, sites, materials, and so on. Methods for the monitoring of construction safety include both manual observation and image measurement. The mainstream image measurement is divided into 2D (acquired using surveillance cameras, fixed cameras) and 3D (acquired using RGB-D sensors, LIDAR) images based on the dimensionality of the captured images [223]. CV techniques such as target detection, target tracking, and action recognition, combined with deep learning algorithms from images or videos, can automatically monitor construction site information to ensure construction site safety and productivity. The traditional image measurement method with a fixed camera position, on the other hand, is incapable of adapting to the complex and changing environment of the construction site. The color of workers’ clothing, the color of the site lighting, and the camera position all have an impact on information acquisition. As a result, the combination of computer vision and robotic automation technology has emerged as a new trend in the construction industry.

Most of the early robots combined robotics and common construction means to replace human labor, and they are commonly used for static manual labor, where the subject position is essentially fixed, such as electric welding [224], bricklaying [225], and assembly facilities [226,227].

Mobile robots based on proximity sensors and CV technology were further developed to overcome the limitations of fixed camera positions [228,229]. Li et al. [230] created a mobile robot with an intelligent lifting system that uses the YOLOv2 algorithm to automate the lifting of large components such as prefabricated floor slabs. Kim et al. [231] created and validated a framework that can automate real-time target detection and predict trajectories in construction using a camera-mounted UAV and the YOLOv3, DNN (S-GAN) algorithm. Wang et al. [232] used a construction waste recycling robot to develop a Faster R-CNN target detection algorithm and a full-coverage path planning algorithm. To ensure worker safety while reducing material waste, this robot can monitor and retrieve nails and screws scattered on the ground in real time. Luo et al. [233] proposed a framework for intelligent pose estimation for various types of construction equipment. Both the Stacked Hourglass Network (HG) and the Cascaded Pyramid Network (CPN) models were found to be more than 90% accurate. Lee et al. [234] created an autonomous mobile robot capable of the real-time monitoring of PPE usage on construction sites. It can inspect unsafe behaviors automatically by utilizing the SLAM algorithm and YOLOv3 to achieve localization navigation and target detection functions.

All of the studies mentioned above strongly support the prospect of combining automated robots with CV technology, which is beneficial in terms of cost savings and efficiency. Currently, certain technical challenges remain to be overcome in the acquisition, training, and analysis of high-quality datasets in complex environments on construction sites.

## 5. HVAC Equipment Monitoring

### 5.1. Thermal Infrared-Image-Based Device Monitoring

Failures in equipment and components can result in abnormalities in a system’s temperature distribution. As a result, the temperature can be used to analyze the operating status of HVAC system equipment and piping, and it is one of the most commonly used indicators in the field of equipment monitoring.

**Figure 16:**
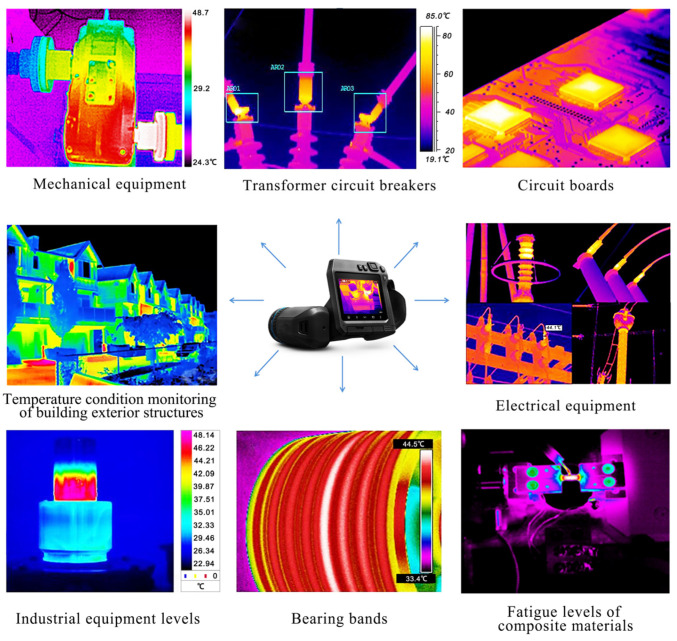
Multiple condition monitoring applications for TIR.

TIR cameras are non-contact condition monitoring instruments that can measure an object’s temperature and dynamic changes from a distance in order to obtain thermal images of equipment or components, and they can then monitor and analyze temperature anomalies in order to alert personnel to ensure timely maintenance and prevent failures. Infrared imaging technology is maturing and is widely used for condition monitoring in a variety of fields, such as machinery monitoring, electrical equipment monitoring, civil structure monitoring, and nuclear industry monitoring (Figure 16) [234,235,236,237,238,239,240].

The HVAC system is complicated, incorporating multiple parts of the refrigeration system, electrical system, and air system, as well as a variety of equipment. The system’s faults are interconnected and affect one another, and the causes are complex and difficult to identify as a whole. Electric motor (IM) failures, mechanical failures, and thermal failures are all common in HVAC systems [241,242,243].

Infrared imaging is widely used for thermal troubleshooting and HVAC system performance monitoring. Using infrared images, researchers have monitored the heat transfer of heat exchanger or condenser fin surfaces, as well as tube walls [244,245]. To monitor the heat transfer performance of air-cooled condensers, Ge et al. [246] used infrared imaging to obtain the condenser’s overall and local surface temperature profiles. They established that the ambient air temperature, natural airflow, and surface defects all have an impact on unit performance. Based on infrared images, Sarraf et al. [247] investigated the effect of steam desuperheating on the performance of a plate heat exchanger as a condenser. The presence of superheated steam at the condenser inlet improved the local heat transfer at lower than saturated wall temperatures. Based on infrared images, researchers have investigated the effect of the number of fins, shape, height, width, and Reynolds number on heat exchanger performance [248,249,250]. The use of shorter continuous corrugated fins in genset air-cooled condensers can improve the heat transfer efficiency [251]. Choosing the appropriate number of fins improves the heat transfer performance of the steam chamber heat sink at low Reynolds numbers. Choosing a larger number of fins improves the heat transfer performance at high Reynolds numbers [252]. TIR-based equipment condition monitoring ensures that the equipment is operating normally and safely, improves system heat transfer efficiency, and lowers maintenance costs.

**Figure 17:**
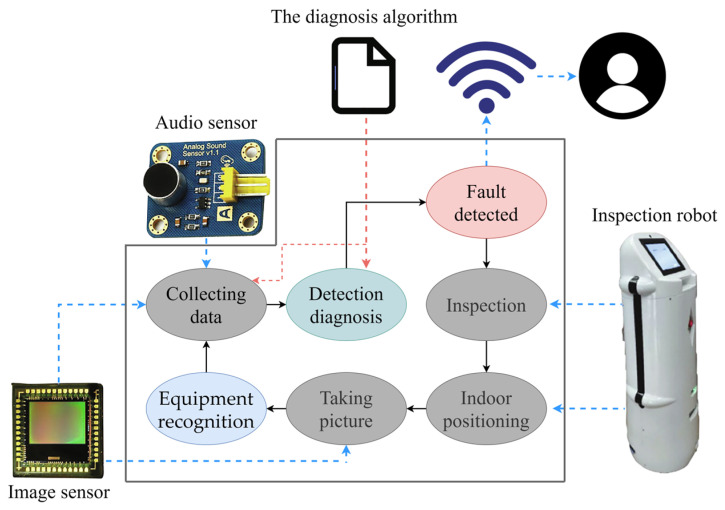
Detection and diagnosis framework based on robotic inspections [253].

He et al. [253] used video and audio signals to develop a non-contact method for fault diagnosis and detection in refrigeration plant rooms based on an inspection robot. As shown in Figure 17, the inspection robot collects video and audio data with an infrared camera, a standard camera, and a microphone. First, an image of the machine room’s equipment is captured using a standard camera at a predetermined location, and the image is classified using the AlexNet convolutional neural network. If the image classification involves a dial, the dial value is read using the image morphology method to determine whether the pipeline is functioning properly. If the image classification involves a pump, the audio sensor is used to determine whether the sound is normal, and an infrared camera is used to acquire a thermal image of the pump. The optical character recognition (OCR) method is used to determine whether the pump is overheating by identifying the maximum temperature of the pump on the infrared image. The relevant experiments were carried out in an air conditioning plant room in Shanghai, China for this study. The results showed that the proposed method can detect mechanical and thermal faults in equipment and piping. However, the method is currently designed and applied primarily for pump and dial faults in refrigeration rooms, and non-contact fault diagnosis methods applicable to other refrigeration room equipment must be further developed.

### 5.2. Visible-Image-Based Device Monitoring

Visible light images combined with video image processing techniques can provide more visual information than infrared thermal images. The monitoring of equipment health using visible light images is a research area that has received a lot of attention in recent years. In particular, progress has been made regarding the problems of frost on the surfaces of heat pumps and condensation on the surfaces of heat exchangers.

#### 5.2.1. Heat Pump Surface Frosting Phenomenon Monitoring

**Figure 18:**
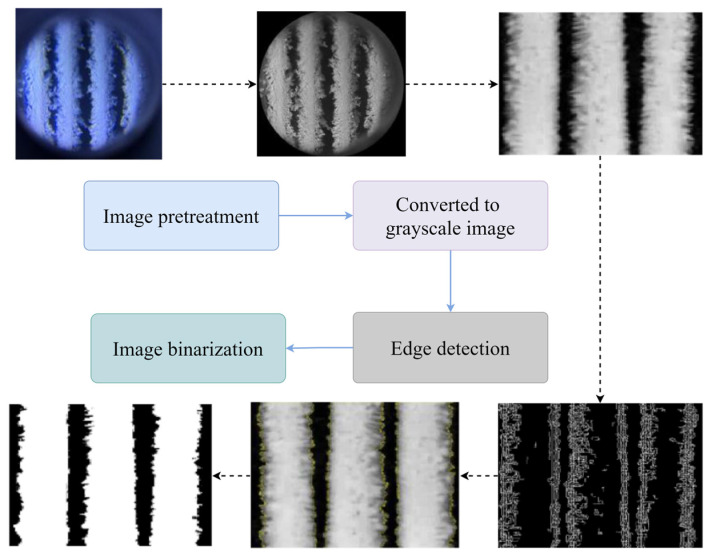
Image processing of frost.

Frost has a significant impact on heat exchangers’ heat transfer performance, and research on frost has focused on the analysis of frost formation mechanisms and characteristics, the simulation of heat exchanger frost characteristics, and defrost control both at home and abroad. Air source heat pump (ASHP) systems have become a common alternative to traditional coal-fired space heating technologies in residential and commercial buildings around the world due to their energy efficiency and environmental benefits. Regular defrosting is required to maintain the safe operating performance of air source heat pumps. Various frost suppression, frost retardation, and defrosting methods have been developed in recent decades, but they are still incapable of avoiding the reduction in energy efficiency caused by incorrect defrosting operations. Energy consumption for defrosting can be significantly reduced and smart defrosting can be achieved by acquiring video or images with high-speed cameras and analyzing the frost growth status using image processing techniques, to explore more precise start–stop control points for defrosting systems (Figure 18).

Early vision studies use the frost thickness, frost coverage, and fractal dimension to quantify the degree of frosting in three dimensions: thickness, area, and density. The system regulates the defrost start–stop state based on whether or not the frost layer’s characteristic parameters reach the predetermined threshold value. In order to observe the variation in the frost layer thickness as well as frost layer growth in a circular tube under various environmental variables, Zhou et al. [254] used a CCD high-speed camera and a microscopic imaging system. The frost layer thickness was calculated using image processing techniques, and the method’s feasibility was verified by comparing the calculated and measured values. Wu et al. [255] investigated the relationship between the distribution of crystals in the frost layer and the thickness of the frost layer using a CCD camera in a parallel-flow evaporator under three different operating conditions. During the frost growth and complete growth periods, the frost crystals accounted for a larger fraction of the frost as they moved closer to the cold surfaces of the fins. Malik et al. [256] proposed a hybrid system with both monitoring and defrosting functions to monitor the evaporator frost thickness in real time and discovered that defrosting when the frost thickness reached an optimal threshold of 6 mm could reduce household refrigerator energy consumption by 10%.

The current stage of frost observation research is focused on improving the original grey value calculation method and creating new frost characteristic parameters in order to achieve more accurate and timely defrost control strategies. Yoo et al. [257] used measured data from an ASHP system to estimate the amount of frost per unit time step and the total amount of frost, to reasonably determine the best defrost start time when system performance decreased. Zheng et al. [258] proposed a new temperature–humidity–image (T-H-I) defrost control method. Non-frosting, moderate frosting, and severe frosting zones were classified using image processing techniques. To evaluate the frost degree, the frost coefficient P was introduced, and the optimal state point was determined and verified: defrosting began at P1 = 0.3 and ended at P2 = 0.05. The T-H-I method’s defrost start–stop control information is more accurate and significantly reduces the false defrost phenomenon. Miao et al. [259] improved the T-H-I method for the characterization of the frost degree in terms of thickness and structure. According to the experimental results, defrosting was performed at a frost thickness of 0.726 mm and a fractal dimension of 2.839, and the optimal state point was terminated when the fractal dimension was reduced to 2.324. The improved T-H-I defrost control strategy improved the accuracy and energy efficiency. Using the characteristic parameter F, Li et al. [260] proposed a method to quantify the degree of frost on the outdoor heat exchanger surface. A series of experiments were carried out to validate the method’s applicability in terms of the shooting angle, imaging pixels, illumination, and outdoor heat exchanger surface temperature, and it was discovered that only ambient illumination affected the method’s detection accuracy in practical applications. Wang et al. [261] proposed surface-source-compensated illumination to improve the new frost detection method, taking into account the effect of illumination variations on the image recognition accuracy. The benchmark illumination surface source was chosen, the influence of the light environment was compensated for with the frost threshold correction coefficient, and an air source heat pump image recognition and frost measurement technology based on light adaptation was developed, which overcame the influence of outdoor light environment variations on the ASHP image recognition and frost measurement. It eliminated the impact of changes in outdoor lighting on the accuracy of ASHP image recognition frost measurement and ensured the accuracy of image recognition and frost measurement.

#### 5.2.2. Indirect Evaporative Cooler Condensation Monitoring

**Figure 19:**
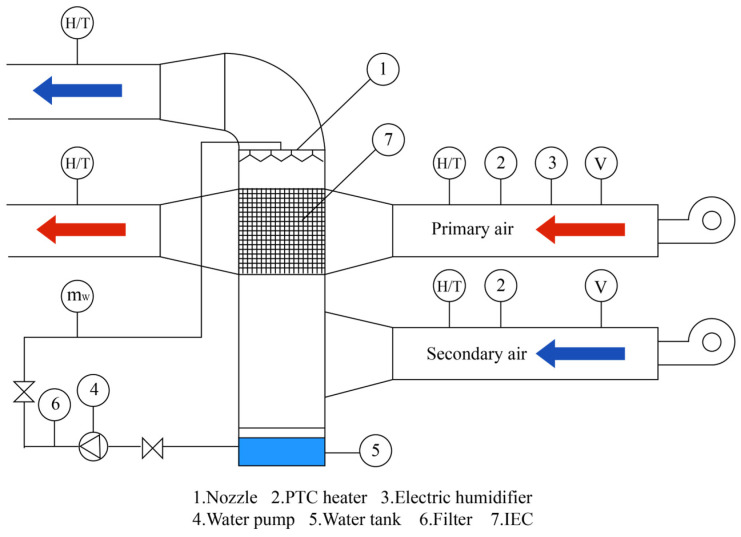
The schematic diagram of the IEC system.

**Figure 20:**
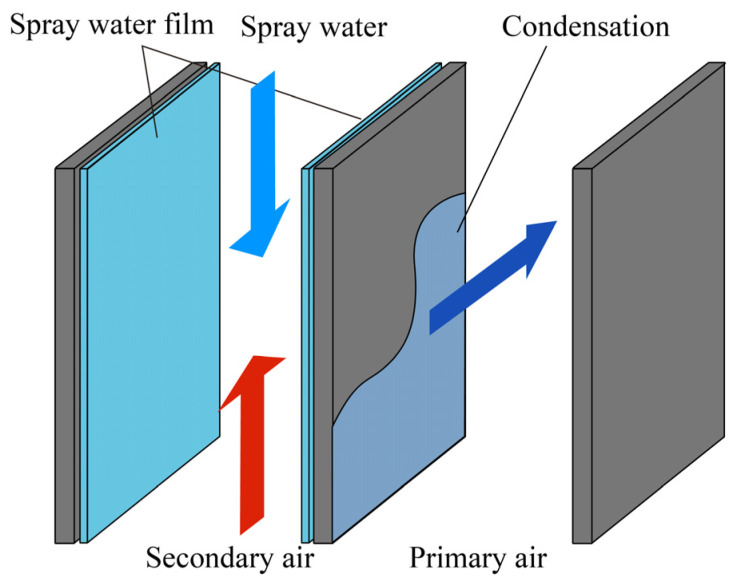
IEC heat and mass transfer mechanism diagram.

The indirect evaporative cooler (IEC) uses the evaporative heat absorption of water to cool fresh air (Figure 19 and Figure 20) with a simple, clean, and efficient structure that has grown rapidly over the decades [262,263,264]. In recent years, the application potential of IEC in hot and humid regions has been investigated, and the hot and humid fresh air in the channels within the primary passages can be cooled to below the dew point, producing condensation. The IEC transforms into a heat recovery device, cooling and dehumidifying at the same time, both of which save energy. With the theoretical study of the condensate film in the primary channel [265,266,267], the visualization study based on CV technology has also received attention.

**Figure 21:**
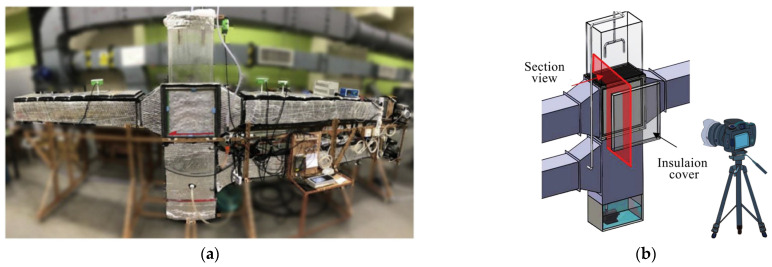
Visualization lab bench [269]. (**a**) Photo; (**b**) 3D schematic diagram.

**Figure 22:**
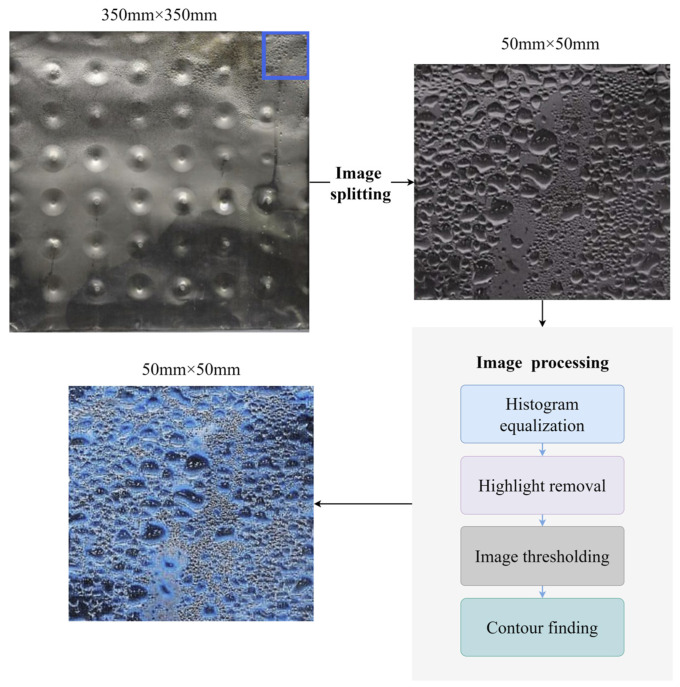
Condensed image processing [269].

The researchers created a visualization experimental bench (Figure 21) and captured images of condensation on the plate surface in the primary channel with a high-speed camera for image processing and analysis. Simultaneously, the IEC performance index was converted based on experimental data provided by the sensing equipment, and a link between condensation and IEC heat and mass transfer performance was established. Meng et al. [268] studied the IEC performance under different inlet conditions by observing the condensation phenomenon in the primary air channel of a fork-flow IEC and obtaining the turning points from no condensation to partial condensation and from partial condensation to full condensation, respectively. The overall performance of the IEC was investigated experimentally under various inlet primary air temperature and humidity conditions. Condensation can raise the outlet primary air temperature and water consumption, reduce the wet bulb efficiency, and increase the total heat transfer by releasing latent heat, according to the findings. Min et al. [269] modified the analytical model of the heat flow density by observing the drop and film condensation area coverage and quantitatively investigated the effects of the inlet primary air temperature, relative humidity, and flow rate on heat transfer by condensation in the IEC, as shown in Figure 22. The experimental results show that as the inlet primary air temperature rises, the area ratios of bead condensation (DWC) and film condensation (FWC) remain relatively stable at 0.4 and 0.6, respectively, while the total heat flow density rises slightly. The primary air relative humidity has a significant effect on the total heat flow density of the plate surface, and when the relative humidity is higher, the FWC increases while the growth rate of the heat transfer coefficient decreases. When the air flow rate is higher, the area ratio of DWC can be increased to improve the condensation heat transfer performance. It has previously been demonstrated that coating the secondary channels of the IEC with a hydrophilic coating can significantly improve the wettability and evaporation efficiency [270]. Min et al. [271] were inspired by this and coated a silicon nanophobic material on the surface of the primary air channel to investigate the effect of the coated hydrophobic coating on the cooling and dehumidification capacity, as well as the heat transfer performance of the IEC, under humid thermal conditions. Using image processing techniques, it was discovered that the hydrophobic surface promoted droplet condensation with smaller droplet diameters. Droplet size reduction and frequent droplet removal could improve the convective heat transfer of treated air flowing on the surfaces of coated panels. IEC energy savings were improved by 8.5–17.2%, indicating potential in dehumidification air conditioning applications.

To achieve two-phase flow heat transfer prediction, the field of multi-phase heat flow research has successfully combined visualization data with machine learning and deep learning. As a result, some researchers have considered combining CV techniques with artificial intelligence algorithms in the future. The dynamic properties of condensate droplets are tracked and analyzed to obtain visualization data such as the nucleation density, droplet growth rate, and average droplet diameter, and datasets are built to combine deep learning, neural networks, and other artificial intelligence algorithms, to train models to establish the link between the visualization properties and heat transfer performance [272,273,274].

The combination of advanced techniques for condensation heat transfer measurements, such as CV technology and artificial intelligence algorithms, is a dependable and cost-effective approach. It eliminates the need for a large number of sensing devices, reduces system errors to some extent, acquires visual features from condensate droplet images only, trains machine learning models and neural network frameworks, and estimates the condensation performance quickly. However, little research has been conducted on the measurement of condensation heat transfer on external surfaces, and the extraction of visualization features, particularly dynamic droplet features, requires further investigation.

## 6. Summary and Outlook

New artificial intelligence technologies have facilitated the widespread use of non-contact measurement methods. Three areas have seen progress: indoor environmental monitoring, outdoor environmental monitoring, and HVAC equipment monitoring.

### 6.1. Indoor Environmental Monitoring

A non-contact measurement method based on infrared and visible images to detect a person’s thermal state from the perspective of the human skin temperature and posture is effective. This method has also been widely used in the fields of sleep state monitoring and on-demand ventilation in recent years.

- The algorithm’s performance in detecting more types of human posture should be improved in the future. Currently, automated quantitative observations of frost and dew condensation are limited to a lateral reflection of condensation through dew coverage.
- The majority of current research is focused on gathering information about the indoor environment. It is necessary to consider combination with control technologies to achieve the real-time automated regulation of indoor environments based on personnel’s thermal status.

### 6.2. Outdoor Environmental Monitoring

The complex and changing outdoor environment can have an impact on pedestrian comfort. It is critical to create high-quality image datasets for urban environmental monitoring, particularly non-contact environmental monitoring in construction scenarios.

- The further integration of SVI, remote sensing images with social media data, weather conditions, human posture, and many other types of heterogeneous urban data should be considered for future use based on the new generation of information technology represented by artificial intelligence (AI), Internet of Things (IoT), digital twin (DT), and inspection robots.

### 6.3. HVAC Equipment Monitoring

The combination of unstructured data (image and audio signals) from HVAC equipment with structured data collected by existing BASs and inspection robots enables the real-time automatic diagnosis of equipment faults.

- To achieve more precise defrosting timing, a variety of frost suppression and frost retardation strategies and defrosting methods are used in conjunction with local conditions. While maintaining indoor thermal comfort, the defrosting process’s energy consumption is reduced, and the unit’s operation is stabilized to reduce the number of defrosts.
- The continued development of an intelligent defrosting strategy based on CV technology to quantify the degree of frosting by inducing new feature parameters from the original image data is necessary.
- It is important to extend the video shooting time and shoot condensation surfaces from multiple camera positions to reduce visualization experimental errors, and to create new CV algorithms that incorporate dynamic droplet features such as the droplet growth rate, shedding frequency, number of droplets merging, and number of shedding, to create more reliable condensation datasets.
- It is also important to generate a generic condensing heat transfer performance prediction model by combining techniques such as CV and AI algorithms such as deep learning.

## Figures and Tables

**Table 1:** Keywords in three areas.

Indoor Environment Monitoring (2013–2023)	Outdoor Environment Monitoring (2013–2023)	HVAC Equipment Monitoring (2003–2023)
computer vision infrared thermal imaging video image processing occupant behavior physiological parameter hot pose cold pose non-contact measurement thermal comfort	outdoor thermal comfort pedestrian thermal comfort street view image street view photographs street-level imager artificial intelligence computer vision visual analytics behavior patterns sky view factor greenway planning urban morphology urban spatial indicators urban environment urban facade color new urban data construction sites construction equipment monitoring robot/robotics visual object detection	infrared thermography infrared thermal imaging equipment health heat exchangers refrigeration fin monitoring fault diagnosis and detection non-contact measurement image robot frosting/frost condensation automatic observation image processing

## Data Availability

Not applicable.
